# Predicting the Time Spent Playing Computer and Mobile Games among Medical Undergraduate Students Using Interpersonal Relations and Social Cognitive Theory: A Cross-Sectional Survey in Chongqing, China

**DOI:** 10.3390/ijerph15081664

**Published:** 2018-08-06

**Authors:** Li Chen, Ruiyi Liu, Huan Zeng, Xianglong Xu, Rui Zhu, Manoj Sharma, Yong Zhao

**Affiliations:** 1 School of Public Health and Management, Chongqing Medical University, Chongqing 400016, China; nameclx@foxmail.com (L.C.); lry981118@foxmail.com (R.L.); zenghuan586@aliyun.com (H.Z.); xianglong1989@126.com (X.X.); 2016111041@stu.cqmu.edu.cn (R.Z.); 2 Research Center for Medicine and Social Development, Chongqing Medical University, Chongqing 400016, China; 3 The Innovation Center for Social Risk Governance in Health, Chongqing Medical University, Chongqing 400016, China; 4 Department of Behavioural and Environmental Health, Jackson State University, Jackson, MS 39213, USA; manoj.sharma@jsums.edu; 5 Health for All, Omaha, NE 68144, USA; 6 College of Health Sciences, Walden University, Minneapolis, MN 55401, USA

**Keywords:** time, gaming disorder, interpersonal relations, self-efficacy, self-control, expectations

## Abstract

*Background*: Computer and mobile games are widely used among undergraduate students worldwide, especially in China. Our objective was to predict the time spent playing computer and mobile games based on interpersonal relations and social cognitive theory constructs (i.e., expectation, self-efficacy, and self-control). *Methods*: The cross-sectional survey was conducted in two medical universities using a sample of 1557 undergraduate students recruited by cluster sampling. The five-point Likert questionnaire was jointly developed by researchers from Chongqing Medical University and Jackson State University. *Results*: Approximately 30% and 70% of the students played computer and mobile games, respectively. The daily times spent by participants on computer games were 25.61 ± 73.60 min (weekdays) and 49.96 ± 128.60 min (weekends), and 66.07 ± 154.65 min (weekdays) and 91.82 ± 172.94 min (weekends) on mobile games. Students with high scores of interpersonal relations but low scores of self-efficacy spent prolonged time playing computer games on weekdays and weekends (*p* < 0.05 for all). Students with low scores of expectation spent prolonged time playing computer games on weekdays (*p* < 0.05). Students with high scores of interpersonal relations but low scores of self-efficacy and self-control spent prolonged time playing mobile games on weekdays and weekends (*p* < 0.05 for all). *Conclusions*: The prevalence and duration of playing mobile games were higher than those of playing computer games among medical undergraduate students in Chongqing, China. This study determined the interpersonal relations, self-efficacy, self-control, and expectation of the students at the time of playing computer and mobile games. Future studies may consider studying the interaction among game-related behaviours, environments, and personality characteristics.

## 1. Introduction

Globally, computer and mobile games are widely played by undergraduate students and have been identified to cause serious public health problems. The use of computer and mobile games is especially prevalent in China. By the end of December 2015, the number of Chinese teenage netizens (18 to 25 years old) reached 287 million, and of these, 66.5% play computer and mobile games [1]. By 2017, China had more than 390 million students playing computer games, accounting for 25.4% of all Internet users [2]. With the development of portable devices, the number of players shifting to mobile phones from computers has increased. Since 2013, 15.6% of users have not played games on their computers [3]. According to a 2015 survey, 90% of teenagers used mobile phones to surf the Internet, and 51.0% of them played mobile games online [1]. Playing computer games for a long period increases the screen viewing time of undergraduate students, causes dry eye syndrome and visual fatigue [4,5] and raises the risk of sedentary behaviour and obesity [6,7,8]. Smartphone overuse can lead to neck, wrist and back pains [9]. Excessive smartphone use at night can also shorten sleep time and lead to stress and depression [10]. In addition, a positive correlation was noted between the severity of Internet addiction and depression in adolescents, but no such relationship was found between time spent using social networks and depression [11]. Moreover, being in a state of negative tension, such as anxiety and depression, can promote the use of smartphones and the Internet and even lead to addiction [12,13].

In the study on Internet or smartphone addiction (computer and mobile games), several models of Internet and smartphone addiction are developed, like the IPACE model of Brand or the model concerning problematic smartphone use of Bilieux [12,14,15]. These models suggest that personality characteristics or psychopathological phenomena (depression and anxiety) significantly influence Internet and smartphone addiction. According to Montag’s research in 2017, the personality characteristics of Internet and smartphone addiction overlap, and the relationship between personality and Internet addiction is closer, with self-efficacy or self-control as potential precursors of addiction [16]. Empathy and life satisfaction are also linked to Internet and smartphone addiction [17]. In addition, a 2015 study in South Korea found that psychopathological phenomenon, such as anxiety, is a risk factor for Internet and smartphone addiction [13]. Preoccupation and conflict are risk factors for smartphone addiction [18]. Depression and attention deficit both play a critical role in Internet addiction [19]. Internet addiction can also predict stress, depression, anxiety, and loneliness [20]. Personality characteristics, such as self-esteem and resilience, also regulate depression and Internet and smartphone addiction [21]. Moreover, a considerable overlap exists between Internet and smartphone addiction [16,22]. This overlap may explain why studying the factors that influence Internet-use disorders (e.g., computer and mobile games) is crucial. Game disorder has progressed and is now officially included in the ICD-11. However, the inclusion of game disorder in the manual is controversial. The theoretical link between game disorders and personality traits and their role as factors of resilience or vulnerability (e.g., self-directedness, extraversion, impulsivity, and empathy) needs clarification.

Time spent on computer and mobile games is affected by many factors. In terms of gender, males are more interested in playing computer and mobile games than females [23]. A Chinese study found that teenage females spend more time on other extra-curricular and leisure activities, whereas teenage males spend more time playing video games [24]. Previous research showed that the length of time spent playing video games varies with age [25]. Smoking and drinking are associated with video games [26,27]. Family situation is also linked to playing computer and mobile games [6], whereby game behavior can enhance family cohesion to a certain extent [28]. Although game behavior is exhibited separately, most players prefer to share activities with friends or parents [28]. This preference may relatively extend game time. Many studies identified factors that influence the amount of time students spend playing computer and mobile games, but few studies focused on interpersonal relations and social cognitive theory constructs at the time of playing computer and mobile games, especially among Chinese medical undergraduate students.

Interpersonal relations and social cognitive theory constructs (i.e., expectation, self-efficacy, and self-control) are associated with game-playing behaviour [28,29,30]. Interpersonal relations measure the relationship between a person and those around him/her. A previous study found that people who play video games possess a good friendship network [28]. The social nature of certain video games can extend playing time whilst allowing players to gain new and other relationships [31]. Social cognition theory is a dynamic and reciprocal model based on the interaction among behaviour, personal factors, and environmental influences [32]. In this theory, expectation, self-efficacy and self-control are the core determinants for achieving a goal [33]. Social cognition theory was applied to study Chinese people for smoking cessation [34] and obesity prevention [35,36]. Expectations include the anticipation of the outcome of a particular action and the importance of the value of these results [37]. Self-efficacy describes a person’s confidence in exhibiting a particular behaviour at a given moment [38]. Self-control describes a person’s capability to regulate behaviour and includes strategies that encourage proximal and distal goal setting and self-rewards [37,38]. Another study found that self-efficacy is a comparatively robust predictor of involvement in massively multiplayer online role-playing game community [29]. In addition, self-control is negatively correlated with online game addiction [30], and students with high self-control spend lesser playing time on video games than those with low self-control. A study on Iranian students revealed that self-control when playing video games differs between male and female students [39].

Despite these efforts, previous studies paid little attention to medical undergraduate students, especially in China. Medical undergraduate students have more academic pressure and longer education years than other college students. Computer and mobile games provide a way for medical undergraduate students to relax. The video game time of medical students and the factors influencing this time may differ from those influencing students of other majors. To the best of our knowledge, this study is the first to use social cognitive theory in predicting the time spent playing computer and mobile games among medical undergraduate students in China. The objectives of this study are to examine the factors influencing students’ behaviours in playing computer and mobile games and the effects of interpersonal relations and social cognitive theory constructs (i.e., expectation, self-efficacy, and self-control) on the time spent by students playing computer and mobile games.

## 2. Materials and Methods

### 2.1. Study Design

A cross-sectional study on medical undergraduate students was conducted in Chongqing in March 2018. Each medical university/college in Chongqing consists of a comparable number of students. A total of 1557 students were chosen from Chongqing Medical University and Chongqing Medical and Pharmaceutical College by cluster sampling. Cluster sampling was used to select the classes in school, and 27 classes (6 classes from Chongqing Medical and Pharmaceutical College and 19 from Chongqing Medical University) were selected from the two medical schools in Chongqing. The 27 classes were from Grades 1 to 3 (10 classes from Grade 1, 9 classes from Grade 2, and 8 classes from Grade 3). Prior to the investigation, we conducted a pilot survey in March 2018, involving 50 students from Chongqing Medical University and Chongqing Medical and Pharmaceutical College. With the pilot survey as basis, the questionnaire was distributed by a trained student helper to students who gave written consent to participate. For the questionnaire distribution, the student helper was trained on the purpose and method of the research and was the one who explained the research objectives to the students. The students who signed the informed consent filled out the questionnaire ‘Study on the video games behaviour of undergraduate students in Chongqing, China’, and the participants did not receive incentives. The students were asked to answer the questionnaire in the classroom within 15–20 min. This study was approved by Chongqing Medical University (Reference Number: 2016001), and the ethical approval includes allowing anonymous surveys of minors and adults with the consent of the class counsellor. Written informed consent for processing personal data was obtained from each participant.

### 2.2. Instruments

Questionnaire refers specifically to the measurement instrument used to obtain games related information in this study. The questionnaire was designed by Chongqing Medical University and Jackson State University researchers. The part on interpersonal relations was developed by research analysts from Chongqing Medical University. The part on social cognitive theory was developed by research analysts from Jackson State University. The questionnaire was translated from English into Chinese. We also checked the readability of the translated questionnaire in the pilot survey. The internal consistency of the total questionnaire was 0.954 (Cronbach’s alpha). The games in this study refer to all multiplayer cooperative or independent games that operate on electronic device platforms, including online and offline variants.

The demographic information in this survey included gender, age (15–18, 19–20, 21–28 years old), ethnic group (Han nationals or Minority), grade level (Grade 1, Grade 2, Grade 3) and without siblings (Yes or No). Questions about living habits were also asked, including smoking (Smoker or Non-smoker) and drinking (Drinker or Non-drinker) preferences. This study measured the daily time spent playing computer and mobile games (on weekdays or weekends) with four questions. The participants were asked to answer the questions in min. We considered the score as a game time value which was zero, instead of not playing games when the self-reported game time was zero. The questions about interpersonal relations and social cognitive theory constructs were as follows: 6 items were used to measure interpersonal relations, 4 items were used to measure self-efficacy, 4 items were used to measure the self-control, and 10 items were used to measure the expectation (see Table 1). The internal consistency of the interpersonal relations was 0.929 (see Table 2). The internal consistencies of the self-efficacy, self-control, and expectation subscales were 0.887, 0.915, and 0.944, respectively.

### 2.3. Data Analysis

Frequencies and percentages were calculated to summarize the distributions of the categorical variables. A *t*-test was employed to compare the differences in the continuous variables between males and females. Generalized linear models were developed using social cognitive theory constructs (i.e., expectations, self-efficacy and self-control), interpersonal relations, healthy habits (smoking and drinking status), gender, age group, grade level, lack of siblings, and nationality as independent variables and time spent playing computer and mobile games on weekdays and weekends as dependent variables. Statistical tests included a two-sided test, and statistical significance was at *p* < 0.05. All data were analyzed using SPSS22.0 for Windows (SPSS Inc., Chicago, IL, USA).

## 3. Results

### 3.1. Characteristics of the Sample

This survey involved 1557 undergraduate students. Of the 1241 persons who answered all the questions, 458 (36.9%) were males and 783 (63.0%) were females. All the participants were 15–28 years old, and the average age of the participants was 19.76 ± 1.30 years old. A total of 89.2% were Han nationals; 10.8%, minorities. Of the 1241 participants, 35.1% were Grade 1, 27.0% were Grade 2, and 38.0% were Grade 3. A total of 43.4% had no siblings, and 56.6% had siblings. Moreover, 6% and 11% were smokers and drinkers, respectively, and 94.0% and 89% did not smoke and drink (see Table 3).

### 3.2. Daily Time Spent Playing Computer and Mobile Games among Undergraduate Students

Participants reported spending an average of 25.61 ± 73.60 min playing computer games per day on weekdays. A total of 898 (72.4%) reported spending 0 min playing computer games on weekdays, and 31 participants reported spending over 180 min playing computer games on weekdays. A total of 206 participants played computer games from 0 to 60 min on weekdays daily.

Participants reported an average of 49.96 ± 128.60 min playing computer games per day on weekends. A total of 853 (68.7%) reported spending 0 min playing computer games on weekends, and 85 participants reported spending over 180 min playing computer games on weekends. A total of 144 participants played computer games from 0 to 60 min, and 101 participants played computer games from 90 to 120 min on weekends daily.

Participants reported spending an average of 66.07 ± 154.65 min playing mobile games per day on weekdays. A total of 387 (31.2%) reported spending 0 min playing mobile games on weekdays, and 66 participants reported spending over 180 min playing mobile games on weekdays. The majority of participants spent between 0 to 60 min (546 participants) and 90 to 120 min (181 participants) playing mobile games on weekdays daily.

Participants reported spending an average of 91.82 ± 172.94 min playing mobile games per day on weekends. A total of 400 (32.3%) reported spending 0 min playing mobile games on weekends, and 151 participants reported spending over 180 min on playing mobile games on weekends. The majority of participants spent between 0 to 60 min (360 participants) and 90 to 120 min (208 participants) playing mobile games on weekends daily (see Table 2).

### 3.3. Descriptive Statistics of Interpersonal Relations and Social Cognitive Theory Constructs

Compared with males, females had a significantly higher mean score of interpersonal relations for playing video games (*p* = 0.041). However, no significant differences between males and females were observed in the mean scores of self-efficacy, self-control, and expectation for playing video games (see Table 4).

### 3.4. Generalised Linear Model Analysis for Factors Affecting the Time Spent Playing Video Games

In multivariable analyses, interpersonal relations, self-efficacy, and expectation were associated with the time spent playing computer games (see Table 4). Male students spent considerable time playing computer games on weekdays (*p* < 0.001) and weekends (*p* < 0.001). Students with a high score in interpersonal relations spent a long time playing computer games on weekdays (*p* < 0.001) and weekends (*p* < 0.001). Students with a high score in self-efficacy spent limited time playing computer games on weekdays (*p* = 0.010) and weekends (*p* = 0.011). Students with a high score in expectation spent limited time playing computer games on weekdays (*p* = 0.003).

### 3.5. Generalised Linear Model Analysis for Factors Affecting the Time Spent Playing Mobile Games

In multivariable analyses, interpersonal relations, self-efficacy, expectations, and self-control were associated with the time spent playing mobile games (see Table 5 and Table 6). Students with a high score in interpersonal relations spent a long time playing mobile games on weekdays (*p* < 0.001) and weekends (*p* < 0.001). Students with a high score in self-efficacy spent limited time playing mobile games on weekdays (*p* = 0.002) and weekends (*p* < 0.001). Students with a high score of self-control spent limited time playing mobile games on weekdays (*p* = 0.017) and weekends (*p* = 0.029).

## 4. Discussion

The aim of this study is to determine the interpersonal relations, self-efficacy, self-control, and expectation of the students at the time of playing computer and mobile games among Chinese medical undergraduates. Our results indicate that high interpersonal relations, low self-efficacy, and low expectations lead to longer computer games. We find also that high interpersonal relationship, low self-efficacy, and low self-control lead to longer mobile game time.

Approximately 30% and 70% of the participants played computer and mobile games, respectively. According to a previous study, roughly 63.7% participants are active players of computer and mobile games [40]. Our study reported high gaming rates among medical undergraduate students. In addition, mobile games are more popular than computer games among these undergraduate students. This finding implies that an increasing number of medical undergraduate students are replacing computer games with mobile games. A Chinese study found that 22% of medical undergraduates play mobile games or read online novels in class. The use of mobile phones is not limited by space and time and can even penetrate the classroom. The heavy and tedious courses in medical colleges may lead to high mobile game usage in class. This situation may be one reason why mobile gamers outnumber computer gamers in this setting. Medical schools should strengthen classroom management and enhance interest in the curriculum. Attracting students’ attention in class should be done as much as possible to improve quality of teaching and reduce use of mobile games.

Medical undergraduate students with high interpersonal relations and low self-efficacy showed increased times spent playing computer games on weekdays and weekends. A previous study found that gamers who spent more time playing computer games display more prosocial behavior [41] and wider friendship networks [28]. Games have gradually become a way of maintaining interpersonal relationships in real life among college students. Extended game time may be due to games’ ability to enhance users’ social attributes in real life. Extending game time may help maintain a good relationship and enhance users’ social attributes in real life. Good social conditions may also further increase game time. If students’ interpersonal communication is low, then they tend to avoid social interaction, and the use of games as a popular social way will be reduced. However, people with social difficulties play games for social comfort [42]. Students with poor relationships may also immerse themselves in a game, which is related to the type of game. People with good relationships may be inclined to choose social games, such as massively multiplayer games. People with poor relationships may be inclined to choose games that make them comfortable to ease social difficulties.

A previous study found that self-efficacy in the real world is negatively related to game addiction [43], which is similar to our finding. Self-efficacy may be enhanced or amplified by environmental encouragement. However, our study marks the first time the relationship between social cognition theory and playing time is tested among a specific segment of the Chinese population. Health education workers can enhance self-efficacy for playing a computer game to reduce screen time among undergraduate students, especially students from higher grade levels. A previous study showed that motivational interviewing to enhance self-efficacy through repetition, reinforcement, and encouragement can strengthen the intrinsic motivations of subjects [44]. Short-term goals and rewards can also improve self-efficacy [45].

Students with low expectation reported spending long times spent playing computer games on weekdays. A previous study found that adolescents’ expectation for health behaviour can influence the establishment of individual trajectories of health [46]. Therefore, teenagers can achieve healthy goals by raising expectations and controlling playing time. Students with high expectations are more health conscious and willing to take the initiative to reduce their playing time and gain health. Their parents or peers can encourage healthy behaviours, and such an action can lead to higher expectations. Building high expectations for playing video games for less than 3 h a day is important for adolescents. Health educators can enhance the expectation for playing video games to less than 3 h a day to decrease the time spent playing computer games. Medical undergraduate students who are also drinkers and smokers reported spending long time playing computer games on weekdays. This finding is supported by previous studies [27,47,48]. Previous studies have linked interpersonal relations with social behaviours such as smoking and drinking, and we have found that interpersonal relations are also linked to gaming behaviours, which may suggest that games are a social tool in some way [49,50].

For mobile games, medical undergraduate students with high interpersonal relations, low self-efficacy, and low self-control reported spending long time playing mobile games on weekdays and weekends. Playing mobile games can enhance friendships among peers. A study based on massively multiplayer online role-playing games showed that social interaction is essential in gaming experience. A mobile phone-based social network is a major tool for college students to socialize with others. This feature may extend the time spent by undergraduate students on mobile games. A previous study found that mental health self-efficacy influences the symptom outcomes of a mobile phone user. This study also linked self-efficacy with reduced depression, anxiety, and stress symptoms and reported that this improved work and social functioning [51]. We found that students with high self-control spent lesser time on mobile games than on computer games. Self-control is negatively correlated with mobile phone use [52], which supports our finding. The current study determined that self-control is negatively associated with mobile games. Students with low self-control are unable to control themselves from playing mobile games. Health education workers can enhance the self-efficacy and self-control of undergraduate students for playing mobile games to reduce time spent playing mobile games.

However, gender, age, ethic group, and grade levels are not significantly associated with the time spent playing mobile games. No space or time limit is found for mobile games, and mobile games are more popular than computer ones for males and females. A 2014 research found that when surfing and playing games become the main functions of mobile phones, males are more likely to use mobile phones than females [24]. In recent years, an increasing number of mobile games aimed at female groups have been developed, such as ‘Love and Producer’, which is popular among young Chinese women. This activity may be the reason why no significant difference was observed between genders in mobile games in this study. Moreover, age is not significantly associated with the time spent playing mobile games. The participants in this study were mostly 18–22 years old. Age is insignificant, possibly due to the concentration of participants in the age group. More age groups should be included to determine the relationship between age and mobile games.

This study has notable limitations. Firstly, the cross-sectional survey data cannot determine the direction of causality. We cannot clarify if constructs precede behaviour. Secondly, the time spent playing games was self-reported by the participants, which may not be accurate for game time. Self-reported game time of zero is probably the average time of the last month of zero, and this finding does not mean the participant has never played a game. This study also does not distinguish between excessive games because no clear standard for excessive game time is available. The self-reported game times may have introduced information and measurement bias. Future studies can collect the time spent playing games with Internet technologies. Thirdly, the questionnaire on interpersonal relations was developed by researchers in Chongqing Medical University. The interpersonal relationship scale was only tested for internal consistency and readability. It lacks wide recognition and use. Fourthly, our study was conducted on medical universities, which do not accurately represent all the undergraduates in China. Future research requires a larger sample size to cover more students with different majors. Fifthly, self-efficacy, self-control, and expectation are strictly focused on computer and mobile games, and this focus may have inflated correlations with self-reported gaming. Future studies may consider studying the relationship between self-efficacy, which is not strictly focused on computer and mobile games, and the time spent playing computer and mobile games. Finally, the types of games are not distinguished in this study, and different types of games may have different playing times.

## 5. Conclusions

The prevalence and duration of playing mobile games are higher than those of playing computer games among medical undergraduate students in Chongqing, China. This study determined the interpersonal relations, self-efficacy, self-control, and expectation on the time spent playing computer and mobile games. This study reported on students’ gender, age, grade level, and drinking and smoking status at the time of playing computer and mobile games. Future studies may consider studying the interaction among game-related behaviours, environments, and personality characteristics.

## Figures and Tables

**Table 1:** Specific problems in interpersonal relationships and social cognitive theory constructs.

Constructs	Specific Problems
Interpersonal relations ^1^	How much do you agree with the following statement? I have good relationship with…
	(1) my classmates, (2) my roommates, (3) everyone around me, (4) my parents, (5) my teachers, (6) anyone. I don’t often have conflicts with people.
Self-efficacy ^2^	How sure are you that you will…
	(1) play computer or mobile games for less than 3 h daily? (2) reduce the time spent playing computer or mobile games, even if you enjoy playing games? (3) reduce the time spent playing computer or mobile games if you have to hand in your homework? (4) reduce the time spent playing computer or mobile games if you have to do something important?
Self-control ^3^	How sure are you that you will…
	(1) set a goal to play computer or mobile games for less than 3 h daily? (2) reward yourself for insisting on reducing the time spent playing computer or mobile games daily? (3) remind yourself to insist on playing computer or mobile games for less than 3 h daily? (4) constantly check progress to make sure you play computer or mobile games for less than 3 h daily?
Expectation	If I play computer games or mobile games for less than 3 h daily, I will … ^4^
	(1) have additional friends, (2) have more spare time, (3) enjoy more, (4) feel more relaxed, (5) be able to study well.
	Which of the following changes are important to you? ^5^
	(6) have additional friends, (7) have more spare time, (8) enjoy more, (9) feel more relaxed, (10) be able to study well

^1^ Response options were “Not At All Agree”, “Slightly Agree”, “Moderately Agree”, “Very Agree”,” Completely Agree”. ^2^ Response options were “Not At All Sure”, “Slightly Sure”, “Moderately Sure”, “Very Sure”, “Completely Sure”. ^3^ Response options were “Not At All Sure”, “Slightly Sure”, “Moderately Sure”, “Very Sure”, “Completely Sure”. ^4^ Response options were “Never”, “Hardly Ever”, “Sometimes”, “Almost Always”, “Always”. ^5^ Response options were “Not At All Important”, “Slightly Important”, “Moderately Important”, “Very Important”, “Extremely Important”.

**Table 2:** Times spent playing games by undergraduate students in Chongqing, China (N, %).

Total Time (min)	Time Spent Playing Computer Game on Weekdays	Time Spent Playing Computer Game on Weekends	Time Spent Playing Mobile Game on Weekdays	Time Spent Playing Mobile Game on Weekends
Mean ± SD	25.61 ± 73.60	49.96 ± 128.60	66.07 ± 154.65	91.82 ± 172.94
0 ^1^	898 (72.4)	853 (68.7)	387 (31.2)	400 (32.3)
(0–30] ^2^	98 (7.9)	52 (4.2)	282 (22.7)	159 (12.8)
(30–60]	108 (8.7)	92 (7.4)	264 (21.3)	201 (16.2)
(60–90]	21 (1.7)	19 (1.5)	21 (1.7)	36 (2.9)
(90–120]	72 (5.8)	101 (8.1)	181 (14.6)	208 (16.8)
(120–150]	2 (0.2)	3 (0.2)	2 (0.2)	14 (1.1)
(150–180]	11 (0.9)	36 (2.9)	38 (3.1)	71 (5.7)
>180	31 (2.5)	85 (6.8)	66 (5.3)	151 (12.2)

^1^ 0 min; ^2^ greater than 0 min is less than or equal to 30 min.

**Table 3:** Demographic characteristics of undergraduate students in Chongqing, China.

Variables	Daily Time Playing Computer Games				Daily Time Playing Mobile Games				Total
	On Weekdays		On Weekends		On Weekdays		On Weekends		
	0 min	>0 min	0 min	>0 min	0 min	>0 min	0 min	>0 min	
	N (%)	N (%)	N (%)	N (%)	N (%)	N (%)	N (%)	N (%)	N (%)
Gender									
Male	109 (8.8)	349(28.1)	114 (9.2)	344 (27.7)	202 (16.3)	256 (20.6)	172 (13.9)	286 (23.0)	458 (36.9)
Female	278 (22.4)	505 (40.7)	287 (23.1)	496 (40.0)	696 (56.1)	87 (7.0)	681 (54.9)	102 (8.2)	783 (63.0)
Age									
15–18 years old	63 (5.1)	460 (12.9)	67 (5.4)	156 (12.6)	158 (12.7)	65 (5.2)	140 (11.3)	83 (6.7)	223 (18.0)
19–20 years old	207 (16.7)	458 (36.9)	215 (17.3)	450 (36.3)	493 (39.7)	172 (13.9)	467 (37.6)	198 (16.0)	665 (53.6)
21–28 years old	117 (9.4)	236 (19.0)	119 (9.6)	234 (18.9)	247 (19.9)	106 (8.5)	246 (19.8)	107 (8.6)	353 (28.4)
Nationality									
Han nationals	334 (23.9)	773 (62.3)	348 (28.0)	759 (61.2)	793 (63.9)	314 (25.3)	753 (60.7)	354 (28.5)	1107 (89.2)
Minority	53 (4.3)	81 (6.5)	53 (4.3)	81 (6.5)	105 (8.5)	29 (2.3)	100 (8.1)	34 (2.7)	134 (10.8)
Grade levels									
Grade 1	139 (11.2)	332 (26.8)	144 (11.6)	327 (26.3)	349 (28.1)	122 (9.8)	327 (26.3)	144 (11.6)	435 (35.1)
Grade 2	109 (8.8)	226 (18.2)	111 (8.9)	224 (18.0)	245 (19.7)	90 (7.3)	222 (17.9)	113 (9.1)	335 (27.0)
Grade 3	139 (11.2)	296 (23.9)	146 (11.8)	289 (23.3)	304 (24.5)	131 (10.6)	304 (24.5)	131 (10.6)	471 (38.0)
Without siblings									
Yes	162 (13.1)	376 (30.3)	175 (14.1)	363 (29.3)	364 (29.3)	174 (14.0)	342 (27.6)	196 (15.8)	538 (43.4)
No	225 (18.1)	478 (38.5)	226 (18.2)	477 (38.4)	534 (43.0)	169 (13.6)	511 (41.2)	192 (15.5)	703 (56.6)
Smoking status									
Smoker	21 (1.7)	54 (4.4)	20 (1.6)	55 (4.4)	31 (2.5)	44 (3.5)	27 (2.2)	48 (3.9)	75 (6.0)
Non-smoker	366 (29.5)	800 (64.5)	381 (30.7)	785 (63.3)	867 (69.9)	299 (24.1)	826 (66.6)	340 (27.4)	1166 (94.0)
Drinking status									
Drinker	36 (2.9)	100 (8.1)	41 (3.3)	95 (7.7)	78 (6.3)	58 (4.7)	70 (5.6)	66 (5.3)	136 (11.0)
Non-drinker	351 (28.3)	754 (60.8)	360 (29.0)	745 (60.0)	820 (66.1)	285 (23.0)	783 (63.1)	322 (25.9)	1105 (89.0)

**Table 4:** Interpersonal relations and social cognitive theory constructs among undergraduate students in Chongqing, China.

Interpersonal Relations and Social Cognitive Theory Constructs	Min	Max	Mean (SD)	Standardized Cronbach Alpha	Males	Females	*p*-Value
Interpersonal relations	0.00	24.00	15.56 (5.40)	0.929	15.98 (5.81)	15.31 (5.14)	0.041 *
Self-efficacy	0.00	16.00	10.36 (4.59)	0.887	10.16 (4.36)	10.49 (4.71)	0.208
Self-control	0.00	16.00	9.13 (4.72)	0.915	8.82 (4.57)	9.31 (4.81)	0.068
Expectation	0.00	40.00	25.00 (9.58)	0.944	24.58 (9.32)	25.24 (9.72)	0.238
Total	-	-	-	0.954			

* Statistically significant (*p* < 0.05).

**Table 5:** Generalised linear model analysis of factors that affect interpersonal relations and social cognitive theory constructs of time spent playing computer games among all participants in Chongqing, China.

Parameter	Time Spent Playing Computer Games on Weekdays			Time Spent Playing Computer Games on Weekends		
	B	SE	*p*-Value	B	SE	*p*-Value
Males vs. Females	39.100	4.186	<0.001 **	79.798	7.214	<0.001 **
19–20 years old vs. 15–18 years old	–15.115	5.728	0.008 *	–27.646	9.872	0.005 *
21–28 years old vs. 15–18 years old	–30.535	7.816	<0.001 **	–56.256	13.471	<0.001 **
Han nationals vs. Minority	5.129	6.293	0.415	–3.666	10.847	0.735
Grade 2 vs. Grade 1	5.68	5.307	0.277	4.514	9.147	0.622
Grade 3 vs. Grade 1	20.917	6.266	0.001 *	17.876	10.801	0.098
Without siblings vs. with siblings	–3.149	3.999	0.431	3.711	6.892	0.590
Smoker vs. Non-smoker	1.288	8.532	0.880	51.145	14.706	0.001 *
Drinker vs. Non-drinker	17.201	6.543	0.009 *	14.158	11.277	0.209
Interpersonal relations and Social Cognitive Theory constructs						
Interpersonal relations	2.358	0.465	<0.001 **	3.211	0.801	<0.001 **
Self-efficacy	–1.619	0.627	0.010 *	–2.756	1.081	0.011 *
Self-control	–0.658	0.560	0.239	–0.878	0.964	0.362
Expectation	–0.892	0.296	0.003 *	–0.881	0.510	0.084

* Statistically significant (*p* < 0.05); ** Statistically significant (*p* < 0.001).

**Table 6:** Generalised linear model analysis of factors that affect interpersonal relations and social cognitive theory constructs of time spent playing mobile games among all participants in Chongqing, China.

Parameter	Time Spent Playing Computer Games on Weekdays			Time Spent Playing Computer Games on Weekends		
	B	SE	*p*-Value	B	SE	*p*-Value
Males vs. Females	6.569	9.264	0.478	19.573	10.314	0.058
19–20 years old vs. 15–18 years old	–8.581	12.676	0.498	–11.780	14.114	0.404
21–28 years old vs. 15–18 years old	–15.332	17.298	0.375	–12.047	19.260	0.532
Han nationals vs. Minority	17.008	13.928	0.222	16.723	15.507	0.281
Grade 2 vs. Grade 1	–8.537	11.746	0.467	–0.442	13.078	0.973
Grade 3 vs. Grade 1	–1.095	13.869	0.937	–1.762	15.442	0.909
Without siblings vs. with siblings	2.453	8.850	0.782	–4.443	9.854	0.652
Smoker vs. Non-smoker	–12.803	18.883	0.498	–5.613	21.025	0.789
Drinker vs. Non-drinker	21.086	14.480	0.145	9.465	16.123	0.557
Interpersonal relations and Social Cognitive Theory constructs						
Interpersonal relations	3.783	1.028	<0.001 **	4.671	1.145	<0.001 **
Self-efficacy	–4.208	1.388	0.002 *	–6.157	1.546	<0.001 **
Self-control	–2.954	1.238	0.017 *	–3.011	1.379	0.029 *
Expectation	–0.490	0.655	0.454	–0.477	0.729	0.513

* Statistically significant (*p* < 0.05); ** Statistically significant (*p* < 0.001).
